# Prevalence and Management of Hypokalemia in Peritoneal Dialysis Patients in Qatar

**DOI:** 10.1155/2019/1875358

**Published:** 2019-12-18

**Authors:** Abdullah Hamad, Mohammed Ezzat Hussain, Shaza Elsanousi, Hanaa Ahmed, Luzvi Navalta, Vimala Lonappan, Fadwa Alali

**Affiliations:** Department on Nephrology, Hamad General Hospital, Doha, Qatar

## Abstract

**Introduction:**

Hypokalemia is common in patients undergoing peritoneal dialysis (PD). It is associated with increased cardiovascular and all-cause mortality. Treatment usually includes oral potassium supplements, which are poorly tolerated. Our aim was to evaluate the prevalence of hypokalemia in PD patients in Qatar and to improve treatment measures.

**Methods:**

All PD patients in Qatar with persistent hypokalemia and on potassium supplement were included. We performed a root cause analysis, and a management pathway was created. We collected data before (Period 1) and after (Period 2) implementation of the new pathway.

**Results:**

A total of 143 patients with a mean age of 54 years (range 21–82 years) were included in the study. Initial results of Period 1 showed hypokalemia in 48 patients (34%); of these, 14 (29%) had hypomagnesemia. Hypokalemia resolved in 10 of the patients after correction of their hypomagnesemia. The remaining 4 patients continued to require potassium supplementation despite correction of their hypomagnesemia. We started spironolactone (25 mg daily) in 13 of the hypokalemia patients. After 3 months, their mean serum potassium level improved from 3.2 ± 0.3 mmol/L to 3.9 ± 0.4 mmol/L (*p* < 0.001), and the prevalence of patients with persistent hypokalemia decreased from 36% to 21% (statistically significant with *p*= 0.006). No episodes of hypotension or hyperkalemia were observed. Only 1 patient developed mild gynecomastia without discontinuation of the medications.

**Conclusion:**

Our study showed that hypokalemia is a prevalent problem in PD patients in Qatar. Hypomagnesemia is a significant contributing factor to hypokalemia in PD and correcting it leads to improvement of hypokalemia. Addition of spironolactone is safe and effective in treating hypokalemia. Implementing a holistic pathway led to a significant improvement in hypokalemia prevalence in PD patients.

## 1. Introduction

Hypokalemia is very common among peritoneal dialysis (PD) patients [1, 2]. It is estimated to affect one-third of all PD patients. Etiology is multifactorial, including poor nutritional intake, diarrhea, and medications, along with ongoing loss to dialysate [3–5]. Hypokalemia in PD patients is associated with increased cardiovascular and overall mortality [5–7].

Treating hypokalemia in PD patients is challenging. Potassium supplements can be poorly tolerated with gastrointestinal side effects [8] or difficulty in the preparation of added potassium to PD fluid [9]. Intravenous potassium, usually given in the emergency department, is inconvenient for patients and expensive for the health system. Alternative options for medication can be prohibitive due to cost, such as eplerenone, which is expensive. Poor nutrition, along with multiple etiologies, is systemic in PD patients, making it unlikely that these patients will consume foods with increased potassium levels. All these factors lead to poor compliance and complicate long-term success.

Most studies have focused on a single approach in treating hypokalemia with mixed results regarding outcomes. Our previous practice in Qatar was similar with individual efforts to treat hypokalemia. Therefore, we created a new multidisciplinary holistic approach with step up plans to tackle all aspects of hypokalemia (dietary counselling and medications that contribute and treat the disease) under a comprehensive protocol. Patient-centered individualized care was most important. Here, we studied the outcomes of this project.

## 2. Quality Improvement Project Goals

The goal of our project was to study the prevalence of hypokalemia in PD patients in Qatar and provide the best management protocol. We aimed to develop a pathway for the comprehensive management of hypokalemia and to also study the effects of the new management pathway to hopefully decrease the number of patients with persistent hypokalemia and decrease emergency room referrals for intravenous potassium infusion and episodes of severe hypokalemia (potassium <2.8 mmol/L).

## 3. Methods

Our quality improvement project was conducted in Fahad Bin Jassim Kidney Center which provided care for all PD patients in the State of Qatar in 2018. Qatar's population recently exceeded 2.7 million, with an estimated 950 patients with end-stage renal disease (ESRD) on dialysis, about 770 patients on hemodialysis, and 180 on PD. As this was a quality improvement project, it was exempted from IRB approval as per our institution policy.

Inclusion criteria included adult patients (≥18 years) on PD therapy for ESRD for ≥3 months with persistent hypokalemia (<3.5 mg/dl) or a continuous need for potassium replacement for ≥3 months despite regular management.

**Figure 1:**
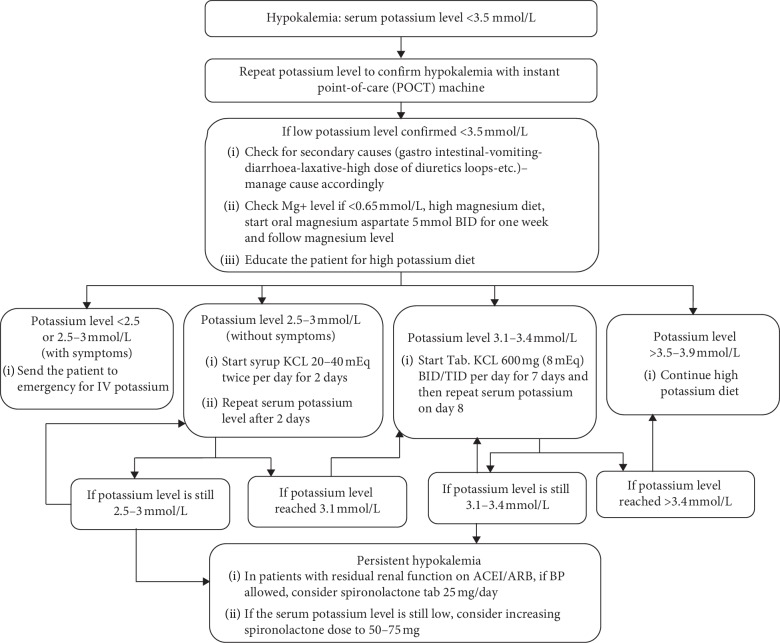
Updated hypokalemia management pathway in peritoneal dialysis patients.

A multidisciplinary team was formed to manage the project. The team consisted of nephrologists, PD nurses, a quality reviewer, and renal dietitian. An initial pathway for hypokalemia management was created based on best practice guidelines. The pathway focused on addressing the causes of hypokalemia, initial intensive dietary management, replacing magnesium deficiency, and exploring using low-dose spironolactone (25–50 mg daily). Eplerenone is not available in our formulary. We used a conservative approach for using spironolactone. Spironolactone was used in patients with high or normal blood pressure, already receiving angiotensin converting enzyme inhibitor or angiotensin receptor blocker (ACE-I or ARB), and with residual renal function of >100 ml/day to maximize its potassium preservation effect. Data were followed for 3 months (Period 1) prior to implementation of our pathway. We re-evaluated our pathway based on the initial data. An updated and final pathway was created to address deficiencies found on the initial approach and to put more focus on creating a process of management daily practice (Figure 1). Patients were followed for an additional 3 months after implementation of the new pathway (Period 2).

## 4. Statistical Analysis

Analysis of the results was performed with Statistical Package for Social Sciences version 17.0 for Windows (SPSS Inc., Chicago, IL). Continuous variables are presented as mean ± SD or median and range, and categorical variables are presented as absolute and relevant frequencies. For comparison between groups, for parametric variables, paired *T* test was used for the comparison, and for nonparametric variable, Chi-squared test was used for the comparison. Probability values of *p* < 0.05 (two tailed) were considered statistically significant.

## 5. Results

**Figure 2:**
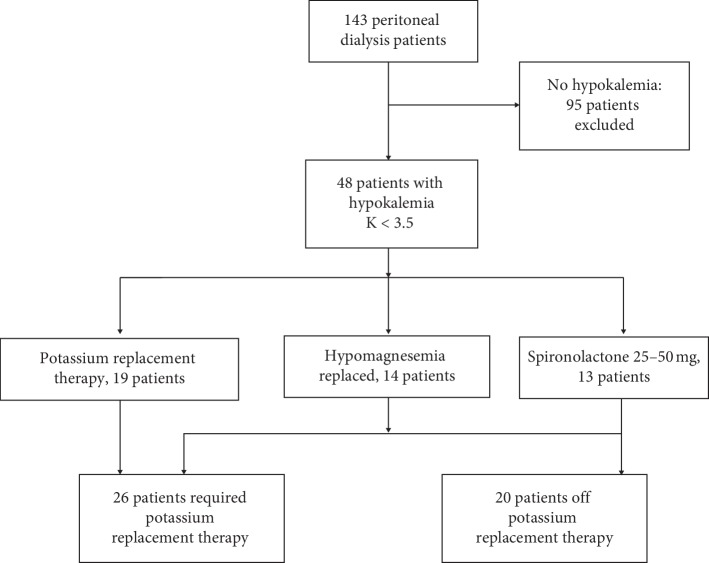
Flowchart of our quality improvement project population and outcome.

We had a stable mean census of 180 PD patients throughout the project duration. A total of 37 patients were excluded due to change in modality, prolonged hospitalization (>1 month), or long travel over 2 months.  Figure 2 summarizes our quality improvement project population and outcomes. We followed and included 143 patients with a mean age of 54 years (range: 21–82 years). During Period 1, there were 48 patients (34%) with hypokalemia or requiring continuous potassium replacement. Demographic and biochemical characteristics of patients with hypokalemia are summarized in Table 1. Root cause analysis of hypokalemic patients revealed that 34 patients had multiple problems, such as poor nutrition, diarrhea, and poor appetite. Fourteen patients (29%) had hypomagnesemia (magnesium level <0.65 mmol/L). All patients with hypomagnesemia were treated with oral magnesium, and their levels were corrected. Ten of 14 (71%) hypomagnesemia patients developed normokalemia without further need for potassium replacement after normalization of their magnesium level. We treated 13 patients who fit our criteria (significant residual renal functions ≥100 ml of urine/day, normal or high blood pressure and already on ACE-I or ARB) with spironolactone. Most patients (12 out of 13) received only 25 mg daily, and one patient received 50 mg daily. Mean serum potassium levels improved from 3.2 ± 0.3 mmol/L to 3.9 ± 0.4 mmol/L after 3 months of spironolactone (*p* < 0.001). Ten patients were able to stop potassium supplement with normal potassium levels with spironolactone support. Only 3 of 13 patients receiving spironolactone continued to require potassium supplements to maintain normokalemia. No episodes of hypotension or hyperkalemia were observed during the time of follow-up with spironolactone. One patient developed mild gynecomastia without discontinuation of the medications. By the end of Period 2, the number of patients that continued to require potassium supplementation for persistent hypokalemia significantly decreased from 48 to 28 (41% improvement) (*p*=0.006). No patients in our overall cohort developed symptomatic or severe hypokalemia (*K* <2.8 mmol/L) requiring emergency intravenous potassium.

## 6. Discussion

Hypokalemia in PD is associated with increased morbidity and mortality. Managing hypokalemia can be challenging. We created a comprehensive pathway to aid in the management of treatment. We based our pathway on analyzing root causes of hypokalemia in a relatively large cohort.

Potassium supplementation has multiple negative problems, such as its bad taste, inducing nausea or even vomiting and epigastric pain, and it is not well tolerated by some patients and the possibility to cause hyperkalemia. We decreased the use of potassium supplements by 41% through use of our alternative pathway that was safe and well tolerated in our project. This led to less number of patients with persistent hypokalemia.

Hypomagnesemia, a well-described cause of hypokalemia, is common in PD patients and is associated with increased mortality [10, 11]. Our study highlights that hypomagnesemia is a common cause of hypokalemia in PD patients and correcting it led to improvement in the potassium level in most of hypokalemic patients. We had 17 patients on proton pump inhibitors (PPI) in our hypokalemic PD cohort. Six of them had hypomagnesemia and 11 did not. Due to the condition of the patients, we could not test if discontinuation of their PPI would improve their magnesium level.

Using spironolactone in PD to treat hypokalemia is gaining more support. It seems not only to tackle urinary potassium loss through the distal tubule, but it also blocks potassium loss from the gastrointestinal track by increasing the apical potassium permeability of the large intestinal epithelium and also blocks loss in the sweat glands and skin [12–14]. Further benefits of spironolactone include reduction of blood pressure and improved survival in dialysis patients [15–17]. Our study did not address other potassium sparing diuretics such as epithelial sodium channel blockers (amiloride or triamterene), but they may have a similar impact on treating hypokalemia in PD patients [18]. A study by Fulop et al. showed that using potassium-sparing diuretics or aldosterone antagonists in PD patients led to a safe improvement in their potassium level with a reduction in the potassium supplementation needs regardless of residual urine output [18]. Additional studies have confirmed the efficacy and safety of spironolactone in treating hypokalemia in PD patients [19–23].

Our experience showed a statistically significant increase of 0.7 mmol/L in the potassium levels. This is similar to the level reported by Amit et al. [19] with a mean potassium increase of 0.5 mmol/L. We opted in our protocol to use spironolactone on PD patients with residual urine output to maximize potassium sparing through the distal tubule and decreased risk of hyperkalemia. Spironolactone improved potassium levels even in anuric hemodialysis patients [24]. However, long-term trials have questioned whether raising the potassium level is a long-lasting effect [25, 26]. The safety of spironolactone has been shown in multiple studies which used it regardless of presence or absence of residual urine output, although they occasionally encountered hyperkalemia [18–23]. None of our patients developed hyperkalemia. Our data confirm this reported safety with only one case of mild gynecomastia. The patient refused to stop spironolactone due to his mild symptoms and preferred it over potassium supplementation.

Finally, despite the mixed ethnic population we have in our cohort (Middle Eastern Arabs, South Asians, and Filipinos), they responded to the protocol similarly. The use of this treatment on this mixed population has not been reported in previously published studies.

## 7. Conclusion

Our study concluded that hypokalemia is a prevalent problem in PD patients in Qatar. Hypomagnesemia is a significant contributing factor to hypokalemia, and correction of hypomagnesemia leads to improvement of the hypokalemia. Adding spironolactone is safe and effective in treating hypokalemia in PD patients with residual kidney functions. Based on these results, we have implemented an algorithm protocol that significantly decreases the number of patients with persistent hypokalemia in our PD population despite different ethnic backgrounds.

## Figures and Tables

**Table 1:** Main demographic and biochemical features in PD patients with persistent hypokalemia.

PD patients	Hypokalemia
Age	55.5 ± 18 years
Sex (female)	23 (47.9%)
Serum Cr (mmol/L)	794 ± 318
Kt/V	2.05 ± 0.39
Creatinine clearance	63.6 ± 18.2 L/week/1.73 m^2^
Urine output	50% of patients had residual urine output with volume 330 ± 450 ml (100–1600 ml); median: 600 ml
BMI	26 ± 4 Kg/m^2^
PD vintage	42 ± 44 months (range: 4–219 months)
	Median: 24 months
Total exchange volume	9.96 ± 3.16 l (range; 6–15.5 L)
PET	High 11 (23%)
	High average 26 (54%)
	Low average 10 (21%)
	Low 1 (2%)
PD modality	APD 18 (38%)
	CAPD 30 (62%)
Phosphorus (mmol/L)	1.38 ± 0.4
Calcium (mmol/L)	2.33 ± 0.15
Albumin (gm/L)	34.1 ± 4
DM	20 (41.6%)
Loop diuretics	15 patients
Hg (gm/d L)	11.1 ± 1.7
Iron sat (%)	28.6 ± 13
Ferritin (ug/L)	612 ± 407
PTH (pg/m L)	318 ± 317
Ethnic	Middle East, 21 (43.5%)
	Philippines, 11 (23.2%)
	South Asia, 16 (33.3%)

## Data Availability

The data used to support the findings of this study are available from the corresponding author upon request per Hamad General Hospital policy.
